# The Latest Elixir Vitæ

**Published:** 1889-08

**Authors:** 


### THE LATEST ELIXIR VITÆ.

The belief that there is, somewhere in nature's vast storehouse, a something, capable of a perpetual regeneration of the physical man, has obtained in all ages, and in all countries, and this something has been the object of deep research by all classes of investigators. The ancient Alchemists sought to distil it from the precious metals, and Bulwer, in "Strange Story," tells of such an elixir, made by an old chap, who figures in the early chapters, and gives, in young Margrave, who is supposed to have gotten hold of it, an illustration of its rejuvinating effects. Margrave, once old and decrepit, is made a blooming youth—but—is devoid of all moral sense; he is simply a splendid animal.—This is significant.

Our readers are doubtless familiar with the story of Ponce de Leon's search for the Fountain, which was to restore his lost youth. Dr. Faustus, doubtless despairing of finding any "elixir"

which would make him "a boy again"—if he ever looked for it, took a short cut to attain his object. He sold his soul and body to the devil for twenty-five years of youth and pleasure, and according to Gœthe, he was duly "signed, sealed and delivered," after a period spent mostly in rioting and debauch.

But it remained for the distinguished and venerable Brown-Sequard to find the genuine article. The alchemists having exhausted the mineral, and the botanists the vegetable, he finds it at last in the animal kingdom! And, Eureka! the world rejoices, and "Dr. Whittier," whose mission, according to his standing declaration in certain St. Louis papers, is to "restore lost manhood," has lost his occupation! The new "elixir" is to supercede, we suppose, all previous efforts to turn back time, and make old men "as good as new;"—crutches are to be relegated to the attic, and all modern Lazaruses are to be made to "get up and walk."

But how strange that the philosopher should have found the philosopher's stone, in the "stone" of a "young animal," and a canine at that! To us there is something indescribably ludicrous in the idea, or the association of ideas;—that a general tonic, or nerve stimulant, was to be found in the testicles of "a young animal." Why the testicles? Like many old men, who, as Dr. Faustus is supposed to have done, sigh for the pleasures of lost youth, and indulge in voluptuous dreams,—we rather apprehend that the philosopher, B.-S. let his mind go out in search of an aphrodisiac;—tradition says he has been experimenting in that direction for twenty years, and he, in reporting, mentions or alludes to the effect on the sexual organs only incidentally,—but in such a way as to warrant the suspicion that that is what he was looking for, and the secret of his great joy. He says that when the fluid was injected into an "old dog" it made him "quite frisky;" and speaking of its effects on himself, says significantly, after enumerating the effect on the bowels, bladder, muscles and brain, "it manifested its power in other unmistakable ways."

The Press and Dr. Brown-Sequard's confreres, have treated him, and his announcement with great respect; though declining for the most part, to accept his statements. This must be due to the great veneration in which he is held, for, had any one else seriously promulgated such a thing, or made such preposterous claims as to personal experience, he would have been laughed to scorn. It is, in our judgemennt not a scientific achievement, but a manifestation of coming dotage, if not of senile insanity.

It is claimed that Dr. Shaw in St. Louis, has detected the tubercle bacilli in a fluid prepared after Brown-Sequard's formula. If this be true, it should raise an insuperable barrier against the practice ever becoming general; but the danger of transmitting disease, or of poisoning your patient by the introduction of ptomains, or of producing blood poison, or thrombi, or what not, are not the only dangers. Suppose B.-S. should be right, after all, and, suppose the "juice of a young dog's testicle," should have the power of rejuvinating the old men; is there not a danger of imparting, at the same time, the elements of the brute nature? of converting your patient, if injected with "lamb juice," into a lamb, goat juice, into a goat, or if that of a dog be used, may it not change your old man, essentially, into an old dog? although it should make him quite frinky? For Bulwer, just quoted, implies in "Strange Story," that the rejuvinating process brought about by an *elixir vitæ* does not extend to the moıal, or intellectual life; only the physical.

Whatever may be the outcome of this sensation scientificially, it cannot fail to have a pernicious effect upon society. We look to see "Brown-Sequard's Elixir" advertised for the restoration of "lost manhood," to see it take the place in the yellow back literature, of "Mormon Elder's Pills" and "Damianua Wafers;" offered to the youth of the country, as well as to the aged, under the name of "Testicline," or or some other "ine" as a sure remedy for that which many youths imagine exists in them—impotence; and there is no estimating the amount of damage already done, especially as such men as retired Surgeon-Generals have given it a quasi endorsement by seriously experimenting with the "elixir."

But, what of the demand for "young animals,"—biped and quadruped, in the possession of the requisite appendages? Is this thing going to effect the price of mutton? or will it aid the sanitary officers in cities in abating the dog nuisance? Meanwhile every "young animal" of whatever genus, who is the proud possessor of "testamentary" or testicular ornamentation, should beware. Dr. Merryman suggests that the suspensory bandage may perhaps become an article of prime necessity with such, and that perhaps a use has here been found for certain "young animals" of the genus *homo*, who part their hair in the middle, and suck the head of a big cane, while obstructing the sidewalks in front of our fashionable churches "mashers" in fact; let the mashers become the mash*ees*.

